# Designer GPCRs as Novel Tools to Identify Metabolically Important Signaling Pathways

**DOI:** 10.3389/fendo.2021.706957

**Published:** 2021-07-20

**Authors:** Jürgen Wess

**Affiliations:** Molecular Signaling Section, Laboratory of Bioorganic Chemistry, National Institute of Diabetes and Digestive and Kidney Diseases, Bethesda, MD, United States

**Keywords:** G protein-coupled receptors, G proteins, type 2 diabetes, obesity, mutant mouse models, DREADD technology, chemogenetics

G-protein coupled-receptors (GPCRs) form a very large family of cell surface receptors that respond to an extraordinary variety of extracellular ligands and sensory stimuli (1). The human genome codes for ~800 distinct GPCR genes, representing ~3-4% of all human genes (2). Approximately 1/3 of all FDA-approved drugs act on one or more GPCRs, indicative of the enormous clinical relevance of this class of receptors (3). Upon binding of extracellular ligands, GPCRs activate distinct classes of heterotrimeric G proteins, which are composed of four major subfamilies, G_s_, G_i_, G_q_, and G_12_ (heterotrimeric G proteins are named after the α-subunits present in the heterotrimeric complex) (4). The receptor-activated α-subunits then modulate the activity of distinct intracellular signaling pathways (4) (also see **Figure 1**).

**Figure 1:**
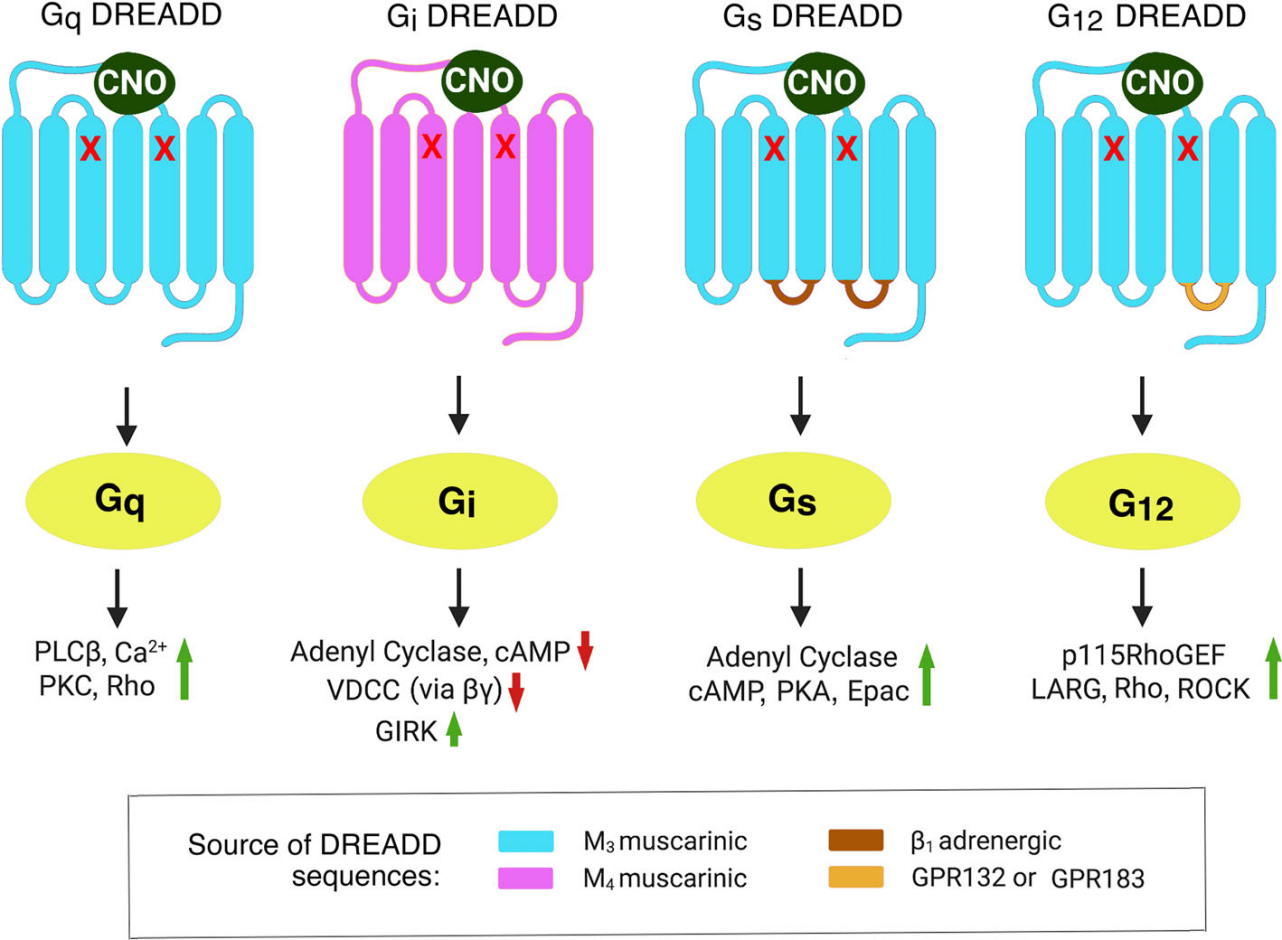
Structural properties of DREADDs able to selectively activate each of the four major subclasses of heterotrimeric G proteins. The DREADDs shown here are all mutant muscarinic acetylcholine receptors that can be activated by CNO with high potency and efficacy. The red x marks indicate point mutations that prevent acetylcholine from activating these designer receptors (5). The G_q_ and G_i_ DREADDs were developed by Armbruster et al. (5). The G_s_ and G_12_ DREADDs were generated in the laboratories of Jürgen Wess (6) and Asuka Inoue (7), respectively. Activated G protein α-subunits stimulate or inhibit distinct intracellular effector enzymes or ion channels. This figure represents a modified version of Figure 1 published in (8). Epac, exchange protein activated by cAMP; GIRK, G-protein-regulated inward-rectifier potassium channel; LARG, Leukemia-Associated RhoGEF; PLCβ, phospholipase Cβ; PKA, protein kinase A; PKC, protein kinase C; RhoGEF, Rho guanine nucleotide exchange factor; ROCK, Rho-associated coiled-coil-containing protein kinase; VDCC, voltage-dependent Ca^2+-^channel.

Like all other cells, metabolically relevant cell types express dozens of different GPCRs (9). However, each individual GPCR is expressed by many other cell types and tissues (9). Moreover, agonist and/or antagonist ligands with high selectivity for a particular GPCR are not available in many cases. For these reasons, it has been very challenging to elucidate the *in vivo* metabolic roles of specific GPCR/G protein signaling pathways operative in a particular cell type.

To circumvent these obstacles, my lab, as well as other research groups, started to employ a chemogenetic approach involving the use of designer GPCRs known as DREADDs (designer receptors exclusively activated by a designer drug) (5). Structurally, the most commonly used DREADDs are mutant muscarinic acetylcholine receptors which, due to the presence of two point mutations in the transmembrane core, show little or no activity in the presence of acetylcholine, the endogenous muscarinic receptor agonist (**Figure 1**). However, muscarinic receptor-based DREADDs can be efficiently activated by a synthetic compound called clozapine-N-oxide (CNO) (5, 6). CNO is otherwise pharmacologically inert, at least when used in the proper dose or concentration range. More recently, CNO derivatives with increased metabolic stability and improved pharmacokinetic properties have been described (10, 11). During the past 15 years, DREADDs that are selectively linked to each of the four major classes of heterotrimeric G proteins have become available (5–7) (**Figure 1**).

Since the development of the first DREADDs in 2007 (5), these new designer receptors have emerged as very useful tools to study GPCR physiology. The *in vivo* use of DREADD technology offers several major advantages that help understand the cellular mechanisms underlying GPCR-mediated metabolic effects. DREADDs with different coupling properties can be expressed in a cell type-specific fashion, for example by generating transgenic mice or by using virus-based delivery techniques. CNO treatment of these mutant mice then leads to the selective stimulation of a particular GPCR signaling pathway only in DREADD-expressing cells. Thus, this approach makes it possible to assess the *in vivo* consequences of activating distinct GPCR signaling pathways in specific cell types. Such studies cannot be performed by traditional pharmacological approaches.

During the past decade, we and other laboratories generated many mutant mouse strains that express different DREADDs in distinct cell types that are critical for maintaining glucose and energy homeostasis [for recent reviews, see (8, 12)]. These include β- and α-cells of the endocrine pancreas, adipocytes, hepatocytes, skeletal muscle cells, and distinct neuronal subpopulations of the hypothalamus. In most cases, CNO treatment of the various DREADD mutant mice resulted in robust metabolic phenotypes. In many cases, the observed phenotypic changes were more pronounced when mice were maintained on a high-fat (obesogenic) diet which causes impaired glucose tolerance and insulin sensitivity, two hallmarks of type 2 diabetes (T2D) (8, 12). To demonstrate that DREADD-mediated responses do not diminish over time, several studies also examined the metabolic effects of chronic CNO treatment [e.g (13–15)].

The metabolic phenotypes observed after CNO treatment of the different DREADD mutant mouse lines led to the identification of several distinct GPCR signaling pathways critical for the maintenance of glucose and/or energy homeostasis (8, 12). For example, studies with DREADD mutant mice suggest that agents able to disrupt hepatocyte G_q_, G_s_, or G_i_ signaling may prove useful to restore euglycemia under pathophysiological conditions associated with enhanced hepatic glucose production (e.g. in T2D) (16–18). Drugs capable of selectively activating G_s_ or G_i_ signaling in adipocytes may prove beneficial to restore impaired energy, lipid, and glucose homeostasis in T2D and obesity (14, 15). Agents capable of activating G_s_ or G_q_ in β-cells are predicted to stimulate glucose-induced insulin release and to promote β-cell replication when applied chronically (6, 13). Compounds that can enhance G_q_ signaling in skeletal muscle tissues could become clinically relevant for stimulating glucose uptake by skeletal muscle in T2D (19). Finally, DREADD studies also strongly suggest that GPCR-based drugs able to modulate the activity of metabolically important neurons of the hypothalamus (e.g. AgRP and POMC neurons) may prove beneficial for the treatment of severe disorders of glucose and energy homeostasis [for a recent review, see (12)].

A major challenge that remains is to identify endogenous GPCRs that are highly expressed in a specific cell type of interest and that display the desired G protein coupling properties, as suggested by the analysis of DREADD mutant mice. Because of the relatively low cellular expression levels of most GPCRs, combined with the lack of highly specific GPCR antibodies, the identification and quantification of GPCR protein levels in specific tissues or cell types is a daunting task. On the other hand, the detection of GPCR transcript levels represents a more straightforward approach and can be achieved by applying qPCR- and RNA-seq-based techniques to cells isolated from humans or animal models (20). Moreover, highly selective agonists or antagonist are not available for a large number of GPCRs. However, the development of such agents will be instrumental for exploiting the new insights gained from the analysis of DREADD mutant mice for therapeutic purposes.

It is also important to verify that the signaling pathways that mediate beneficial metabolic effects in mice are conserved in human tissues. To address this issue, follow-up studies with human primary cells are recommended as a key first step. It has been shown that GPCR expression levels/profiles can undergo significant changes under altered metabolic states and/or pathophysiological conditions [see, for example (14)]. This phenomenon needs to be taken into account during the development of novel ligands aimed at targeting specific GPCR signaling pathways for therapeutic purposes. It should also be noted that activated DREADDs can recruit β-arrestins (21, 22), raising the possibility that DREADD activation may also cause changes in cell metabolism *via* β-arrestin-dependent pathways. For this reason, phenotypic analysis of mutant mice that express distinct DREADDs in specific celll types that lack β-arrestins (23) are likely to facilitate the interpretation of *in vivo* DREADD studies.

In conclusion, the use of DREADD technology has greatly advanced our knowledge about the important metabolic roles of specific GPCR signaling pathways in maintaining proper glucose and energy homeostasis *in vivo*. It is likely that the novel insights gained from these chemogenetic studies will eventually lead to the development of novel classes of drugs that will prove highly efficacious for the treatment of several severe metabolic disorders.

## Author Contributions

The author confirms being the sole contributor of this work and has approved it for publication.

## Funding

The author’s own research cited in this article was supported by the Intramural Research Program of the NIH, NIDDK, Bethesda, Maryland, USA.

## Conflict of Interest

The author declares that the research was conducted in the absence of any commercial or financial relationships that could be construed as a potential conflict of interest.
